# Identification of Transcription Factors of *GmHPL* Involved in Modulating Pathogen Stresses in Soybean

**DOI:** 10.3390/plants15010054

**Published:** 2025-12-24

**Authors:** Yaqi Wang, Wenhuan Lyu, Shuguang Li, Mengmeng Fu, Xiwen Yu, Zhixin Zhao, Shanshan Hu, Haifeng Xu

**Affiliations:** 1 Huai’an Key Laboratory for Agricultural Biotechnology, Key Laboratory of Germplasm Innovation in Lower Reaches of the Huaihe River, Ministry of Agriculture and Rural Affairs, Huaiyin Institute of Agricultural Sciences of Xuhuai Region in Jiangsu, Huai’an 223001, China; yqwang_01@126.com (Y.W.);; 2 Soybean Research Institute, National Center for Soybean Improvement (Nanjing), Key Laboratory for Biology and Genetic Improvement of Soybean (General), Ministry of Agriculture and Rural Affairs, National Key Laboratory of Crop Genetics and Germplasm Enhancement, Nanjing Agricultural University, Nanjing 211800, China

**Keywords:** hydroperoxide lyase, yeast one-hybrid, transcription factor, pathogen stresses

## Abstract

As an important branch of the lipoxygenase (LOX) metabolism pathway, hydroperoxide lyase (HPL) is involved in regulating plant development and defense responses. However, the upstream regulatory mechanism of HPL remains unclear in soybean. In the present study, by analyzing the upstream promoter region of the *GmHPL* gene, *cis*-elements such as MYB motifs, G-box motifs, ERE motifs and W-box motifs were predicted, which were related to the stress response. Yeast one-hybrid was employed and two transcription factors were identified, GmERF36 and GmILR3. The orthologs of ERF36 and ILR3 in Arabidopsis were involved in pathogen stress. A dual-luciferase reporter assay verified the yeast one-hybrid results and indicated that GmERF36 and GmILR3 suppressed the expression of the GmHPL protein. The qRT-PCR results indicated that *GmHPL* and *GmERF36* initially displayed inverse expression patterns within 24 h after *Colletotrichum truncatum* treatment (*GmERF36* was upregulated while *GmHPL* was downregulated); then, both of them were upregulated before decreasing. The results indicated that the response of *GmHPL* to pathogen stress partially depended on *GmERF36*. Our study gives rise to new insights into the upstream regulatory network of the *GmHPL* gene.

## 1. Introduction

Soybean is not only an important oil and protein crop but also a key component of animal feed. Diseases are a critical factor affecting soybean yields and quality. It is crucial to address disease challenges by examining the plant’s innate defense mechanisms—for example, identifying disease-resistant genes, elucidating the mechanisms of disease resistance, and employing molecular breeding techniques for targeted genetic improvement. The lipoxygenase (LOX) pathway is one of the most widespread lipid-metabolic pathways in living organisms. It oxidizes polyunsaturated fatty acids such as linoleic acid and linolenic acid to generate conjugated diene compounds, playing a significant role in plant and animal growth, development, and responses to biotic and abiotic stresses [1]. Different plants exhibit distinct LOX characteristics, primarily classified into two types: 9-LOX, which adds oxygen at the ninth carbon atom, and 13-LOX, which adds oxygen at the 13th carbon atom [2]. The oxidized products enter multiple branching pathways, undergoing further oxidation. The most extensively studied are the allene oxide synthase (AOS) pathway and the hydroperoxide lyase (HPL) pathway, ultimately producing a series of derivatives collectively termed oxylipins [3,4,5]. These oxylipins participate in plant growth and development (e.g., pollen development, seed maturation) and defense responses (e.g., against insects and pathogens).

Hydroperoxide lyase (HPL) catalyzes the conversion of hydroperoxides into short-chain aldehydes and corresponding long-chain ω-oxo acids, ultimately forming volatile aldehydes and alcohols, known as green leaf volatiles (GLVs) in plants [6]. HPL was first discovered in banana fruits [7] and first named hydroperoxide lyase in watermelon seedlings [8]. HPL specifically catalyzes 13S-hydroperoxides but not 9S-hydroperoxides in soybean [9]. When soybean plants suffer from mechanical damage or pathogen stress, unsaturated aldehydes in GLVs have been shown to inhibit the growth of fungi such as *Colletotrichum truncatum*, *Rhizoctonia solani*, and *Sclerotium rolfsii*. (E)-2-hexenal, (E)-2-nonenal, and (Z)-3-nonenal suppress the growth of *R. solani* and *S. rolfsii* at gaseous concentrations above 35 μM, while higher concentrations are required to inhibit *C. truncatum*. (E)-4-hydroxy-2-nonenal is the most potent inhibitory aldehyde, effectively suppressing fungal growth when added to culture media, although its gaseous form has minimal effects [10]. When plants are damaged by herbivores, the induced GLVs can prevent further damage by insects. These volatiles are also released into the air, signaling neighboring leaves to activate a series of defense responses [11]. However, to date, there are relatively few studies on the regulatory mechanism of HPL. Therefore, research on the upstream transcription factors of HPL can strengthen our understanding of the regulatory network of plant resistance to insects and diseases.

The APETALA2/Ethylene Responsive Factor (AP2/ERF) supergene family is involved in many important processes of plant development and stress, which consist of growth and development, the accumulation of secondary metabolites, the synthesis and metabolism of hormones, and abiotic and biotic stresses [12]. The AP2/ERF gene family can be divided into three subfamilies: APETALA2 (AP2), Ethylene Responsive Factor (ERF), and RELATED TO ABSCISIC ACID INSENSITIVE 3/VIVIPAROUS 1 (RAV) [13]. The AP2 subfamily contains two AP2 conserved domains; the ERF subfamily contains one AP2 conserved domain; and RAV contains one AP2 conserved domain and one B3 conserved domain. Mostly, ERFs specifically bind to the GCC-box and/or dehydration-responsive element/C-repeat (DRE/CRT) *cis*-acting elements to regulate ethylene (ET)-inducible pathogenesis-related (PR) genes and abiotic stress-inducible genes [14]. There are about 160 genes containing ERF domains, distributed across 20 chromosomes, identified in soybean, and they are clustered into eight groups [12].

The basic helix–loop–helix (bHLH) proteins constitute a large and important family of transcription factors that are widely present in eukaryotes. These proteins are characterized by their conserved basic helix–loop–helix structural domain. They play crucial roles in signal transduction, growth and development, and stress responses in plants [15]. The domain comprises approximately 60 amino acids and consists of two functionally distinct regions: a basic region that mediates DNA binding and a helix–loop–helix (HLH) motif that facilitates protein dimerization [16]. At present, the bHLH gene family has been reported in Arabidopsis [17], rice [18], Chinese cabbage [19], Tartary buckwheat [20], eggplant [21], and so on. A total of 340 non-redundant bHLH genes have been identified in soybean, categorized into 24 subfamilies within 15 major groups [22].

In our previous study, GmHPL was involved in the defense response to biotic stresses [23]. However, the upstream regulatory mechanism of GmHPL remains unclear. In this study, we analyzed the *cis*-elements of the *GmHPL* promoter and identified transcription factors through yeast one-hybrid screening and dual-luciferase transient expression. qRT-PCR was performed to show the temporal patterns of *GmHPL* and transcription factors under pathogen stresses.

## 2. Results

### 2.1. Characterization of GmHPL Promoter

**Figure 1:**
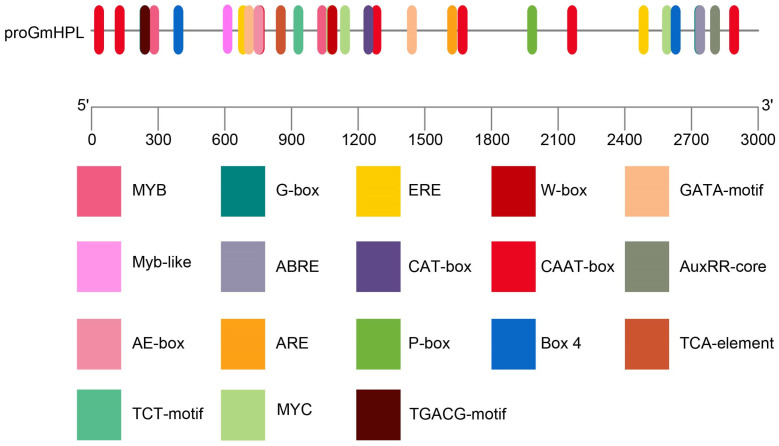
Prediction of *cis*-elements in the *GmHPL* promoter.

A region 3000 bp upstream of the *GmHPL* gene was analyzed via the Plant CARE website (https://bioinformatics.psb.ugent.be/webtools/plantcare/html/ accessed on 10 March 2024). The putative *cis*-elements are not only involved in growth and development, CAAT-boxes, and CAT-boxes but also environmental factor response elements and plant hormone responses (Figure 1). The GATA motif, TCT motif, BOX 4, and AE-box are related to light responsiveness. TCA-elements, ABRE, the P-box, the TGACG motif, the ERE motif, and the AuxRR core are related to hormone responses. The MYB motif binds to MYB transcription factors and functions in plant growth and stress responses. The G-box motif is involved in many important pathways—photomorphogenesis, growth, development, and biotic and abiotic stresses—and is recognized by bHLH and bZIP transcription factors. The ERE motif mediates the transcriptional responses of plants to the ethylene hormone and is recognized by AP2/ERF transcription factors. The W-box serves as the “universal cipher lock” for plant defense and stress responses, while WRKY transcription factors act as commanders possessing the matching “keys”. The results indicated that *GmHPL* may play a role in the response to hormones and stresses during growth.

### 2.2. Two Transcription Factors of GmHPL Were Identified Through Yeast One-Hybrid Assay

A sequence of approximately 2800 bp upstream of the ATG was selected as the promoter of *GmHPL*. Since there are multiple BstbI restriction sites within −1032~−1524 bp in the promoter of *GmHPL*, which makes restriction enzyme digestion verification impossible, this segment was deleted. The prediction of *cis*-elements within the region yielded several CAAT-box motifs, an ARE motif, and a P-box motif. They did not appear to be central to this study. The remaining part of the promoter was divided into two parts: −1524~−2759 bp as GmHPL-pro1 and −1~−1032 bp as GmHPL-pro2.

For self-activation testing, pAbAi-GmHPL-pro1 and pAbAi-GmHPL-pro2 recombinant vectors were transformed into yeast competent cells, which were then spread on SD/-Ura plates containing different concentrations of AbA. The results showed that both pAbAi-GmHPL-pro1 and pAbAi-GmHPL-pro2 grew normally on SD/-Ura plates, indicating no toxicity. However, pAbAi-GmHPL-pro2 grew on SD/-Ura plates containing 900 ng/mL AbA, suggesting that pAbAi-GmHPL-pro2 exhibited self-activation and could not be used for subsequent screening experiments. pAbAi-GmHPL-pro1 did not grow on SD/-Ura plates containing 200 ng/mL AbA, indicating that pAbAi-GmHPL-pro1 exhibited slight self-activation and could be used for subsequent screening experiments (Figure S1).

Using pAbAi-GmHPL-pro1 as the bait, a yeast one-hybrid screen was performed against the constructed soybean whole-tissue yeast library. After primary screening and secondary screening, 64 positive clones were obtained on the SD/-Ura plates containing 200 ng/mL AbA (Figure S2). Then, the clones were subjected to colony PCR and sequencing, resulting in 56 sequencing results. The gene IDs and functional annotations are shown in Table 1. Based on the functional annotations, 13 genes were selected for one-to-one validation, with their gene IDs listed as follows: *Glyma.05G063500*, *Glyma.02G016100*, *Glyma.15G077100*, *Glyma.12G226600*, *Glyma.04G103900*, *Glyma.17G145300*, and *Glyma.13G236500* belong to AP2 superfamily and may bind to the ERE motif; *Glyma.06G266800*, *Glyma.12G136300*, and *Glyma.13G286700* serve as members of bHLH transcription factors and may bind to the G-box motif; *Glyma.17G237900* contains a Myb-like domain that can bind to Myb-like motifs; *Glyma.15G003300* and *Glyma.14G185800* are two members of the WRKY family and may bind to the W-box.

**Figure 2:**
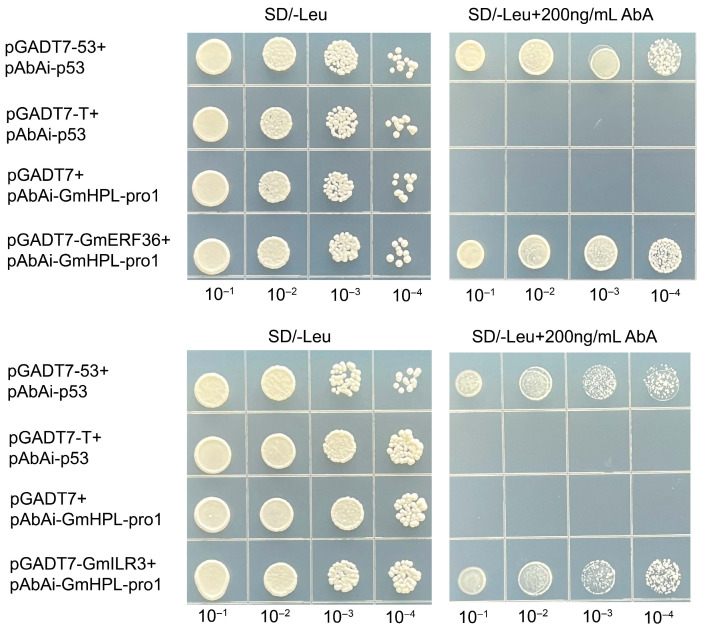
Yeast one-hybrid assay to detect interactions between *GmHPL* promoter and GmERF36 and GmILR3. The yeast strain of pGADT7-53+pAbAi-p53 was a positive control. The yeast strain of pGADT7-T+pAbAi-p53 or pGADT7-T+pAbAi-GmHPL-pro1 was the negative control. The yeast strains of pGADT7-GmERF36+pAbAi-GmHPL-pro1 and pGADT7-GmILR3+pAbAi-GmHPL-pro1 were the experimental groups. All yeast strains were selected on SD/-Leu media at different Aureobasidin A (AbA) concentrations (0 and 200 ng/mL). The numbers (10^−1^, 10^−2^, 10^−3^, and 10^−4^) at the top represent the dilution ratios of the yeast solution.

The constructed recombinant plasmid of the above-mentioned genes was co-transformed with pAbAi-GmHPL-pro1 into yeast competent cells, respectively. On SD/-Leu medium, both negative and positive controls grew at 0 ng/mL AbA, while, at 200 ng/mL AbA, the negative control failed to grow and the positive control grew normally. Consequently, only yeast co-transformed with pGADT7-Glyma.05G063500 and pGADT7-Glyma.13G286700 (and pAbAi-GmHPL-pro1) exhibited normal growth on SD/-Leu medium supplemented with 200 ng/mL AbA. This result demonstrated that *Glyma.05G063500* and *Glyma.13G286700* interacted with the *GmHPL* promoter (Figure 2). According to the functional annotations, *Glyma.05G06350*0 encodes an ethylene-responsive transcription factor 5, which belongs to the AP2 superfamily, and *Glyma.13G286700* encodes an transcription factor ILR3, which belongs to the helix–loop–helix DNA-binding domain superfamily. Based on the protein annotations, *Glyma.05G063500* was named *GmERF36* and *Glyma.13G286700* was named *GmILR3*.

### 2.3. GmERF36 and GmILR3 Inhibited the Protein Levels of GmHPL

**Figure 3:**
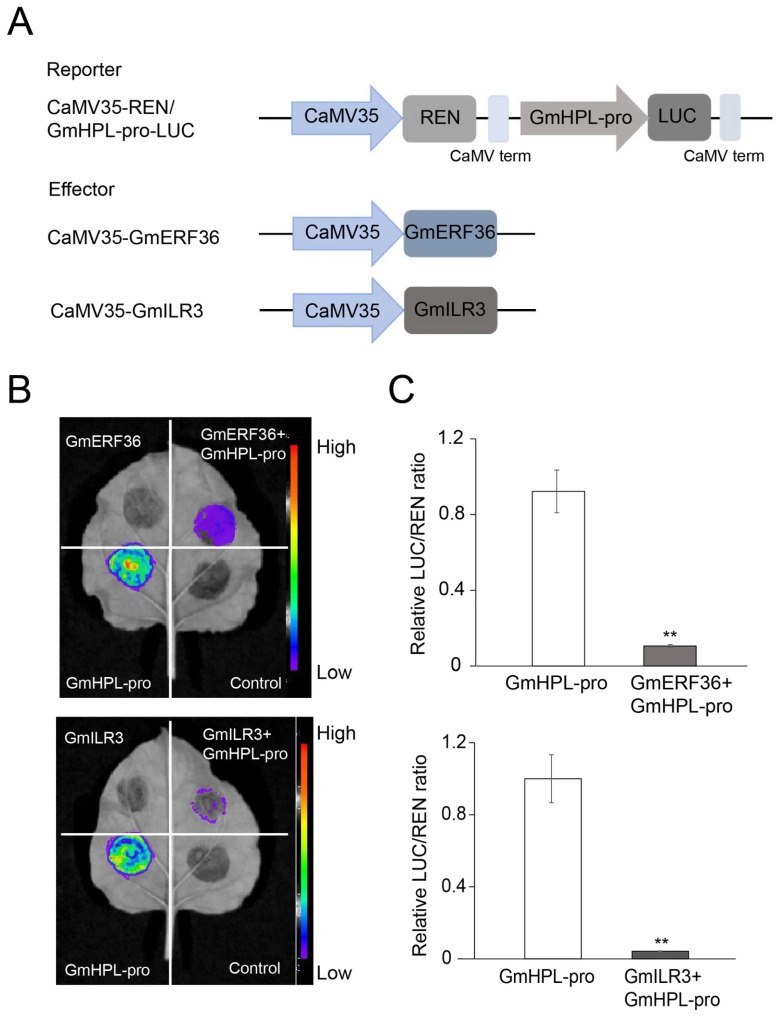
GmERF36 and GmILR3 inhibit the protein levels of GmHPL. (**A**) Schematic diagram of reporter and effector vectors used for dual-luciferase assays. (**B**) Representative dark-field images of transiently expressed LUC-empty vector and 35S-GmERF36 (GmERF36); LUC-empty vector and 35S-GmILR3 (GmILR3); GmHPL-pro-LUC (GmHPL-pro); 35S-GmERF36 and GmHPL-pro-LUC (GmERF36 + GmHPL-pro); 35S-GmILR3 and GmHPL-pro-LUC (GmILR3 + GmHPL-pro); and LUC-empty vector and 35S-empty vector (control) in tobacco leaves, showing GmERF36 and GmILR3 suppression of GmHPL. (**C**) The corresponding relative LUC/REN activity ratio. ** indicates significant differences at *p* < 0.01.

To further verify the interactions of GmERF36 and GmILR3 with the promoter of *GmHPL*, we performed a dual-luciferase assay. The effector constructs, 35S-GmERF36 and 35S-GmILR3, and the reporter construct, GmHPL-pro-LUC, were introduced into the *Agrobacterium tumefaciens* strain EHA105 (Figure 3A). Then, the effector and reporter were transiently co-transfected into tobacco leaves to examine the LUC and REN intensity. The LUC-empty vector and 35S-GmERF36, the LUC-empty vector and 35S-GmILR3, and the LUC-empty vector and 35S-empty vector served as the negative controls, and the reporter alone served as the positive control. As a result, LUC activity and the ratio of LUC/REN activity in tobacco leaves transfected with 35S-GmERF36 and GmHPL-pro-LUC were markedly lower than those in tobacco leaves transfected with GmHPL-pro-LUC, and the same results were found for 35S-GmILR3 and GmHPL-pro-LUC (Figure 3B,C). The original LUC activity and REN activity of GmHPL-pro-LUC, 35S-GmERF36 and GmHPL-pro-LUC, and 35S-GmILR3 and GmHPL-pro-LUC are shown in Figure S3. Notably, GmERF36 and GmILR3 inhibited the protein levels of GmHPL.

### 2.4. Expression Level of GmHPL Induced by C. truncatum Was Partially Dependent on GmERF36

To conclusively confirm that the expression level of *GmHPL* was regulated by *GmERF36* and *GmILR3*, we analyzed the expression levels of *GmHPL*, *GmERF36*, and *GmILR3* in soybean leaves infected with the pathogen. When soybeans had developed five to six trifoliate leaves, *C*. *truncatum* mycelia were homogenized and diluted with sterile water to a concentration of 2 g/L. The suspension was evenly sprayed onto the leaf surfaces. Leaf samples were collected at 0, 12, 24, 48, and 72 h post-inoculation, with three biological replicates per time point. Each repeated leaf was obtained from three individual plants. The primers used for qRT-PCR were shown in Table 2.

**Figure 4:**
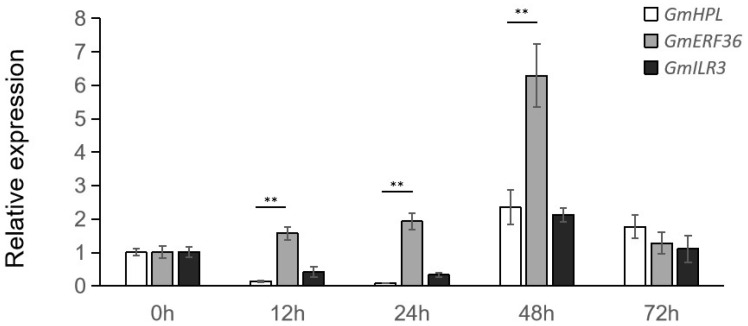
Relative expression level analyses of *GmHPL*, *GmERF36*, and *GmILR3* in Williams82 at different times post-inoculation with *C. truncatum*. Student’s *t*-test was used for the comparisons of three biological replicates, and ** indicates differences at *p* < 0.01.

The results showed that the expression levels of *GmHPL* and *GmILR3* were reduced 10-fold and 2.5-fold, respectively, while that of *GmERF36* increased twofold following treatment with *C. truncatum* (Figure 4). This confirmed that *GmERF36* acted as a negative regulator of *GmHPL* under fungal treatment.

Between 48 and 72 h post-treatment, the expression levels of *GmHPL* and *GmILR3* initially increased approximately twofold and then decreased (Figure 4). In contrast, the expression of *GmERF36* rose sixfold sharply first before declining. These dynamic expression patterns suggested that the regulatory mechanism governing *GmHPL* expression was complex. *GmHPL* was likely modulated by other transcription factors besides *GmILR3* under pathogen treatment.

**Table 2:** Primers used for qRT-PCR in this study.

Primer Name	Forward Primer (5′—3′)	Reverse Primer (5′—3′)
GmActin-qRT	GGTGGTTCTATCTTGGCATC	CTTCGCTTCAATAACCCTA
GmHPL-qRT	CTTCCTCGTCGGTGGAAACT	CCGTAGGAGTTGAAGCCCAG
GmERF36-qRT	GACCTCCTCGAACCCGAAAT	CGATTAGCAGCGACGGTTTC
GmILR3-qRT	GTTGATCGACGACGACGTTAT	TTAGGCCATCAGAATCCCCA

## 3. Discussion

In our previous study, *GmHPL* was involved in the defense response to biotic stresses [23]. However, the upstream regulatory mechanism remained unclear. In the present study, two transcription factors of *GmHPL* were identified and simple functional analyses were conducted.

By analyzing the promoter of the *GmHPL* gene, we found that it contained light response elements (GATA motif, TCT motif, BOX 4, and AE-box), hormone response motifs (TCA element, ABRE, P-box, TGACG motif, ERE motif, and AuxRR core), and environmental factor response elements (MYB motif, Myb-like motif, G-box, ERE motif, and W-box). In Arabidopsis, high-light and wounding treatment induced stronger expression levels of *HPL* [24]. In rice, leaf lesions were light-induced in the HPL deletion mutant *hpl3-1*. According to Plant CARE, the TGACG-motif regulatory element is involved in MeJA responsiveness, and it has been verified in many plants. In our previous study, *GmHPL* was notably induced by exogenous MeJA [23]. In barley leaves, the accumulation of HPL-related aldehydes increased under MeJA treatment [25]. The MYB motif can be activated by abiotic and biotic stresses and interact with the binding sites of the corresponding transcription factors [26,27,28]. The ERE motif was elucidated to bind to ERF transcription factors involved in the ethylene pathway [29]. The W-box motif was verified to bind to WRKY11 in peach fruit functioning in cold storage [30]. The G-box motif was reported to bind to bHLH transcription factors involved in several stress responses [31].

Although extensive studies have revealed that HPL plays a pivotal role in the stress response across species, few studies on upstream regulatory networks have been reported. The *PmF-box1* gene was overexpressed in Arabidopsis and downregulated LOX-HPL pathway genes, leading to a marked reduction in hexanal production [32]. However, no direct interaction between PmF-box1 and LOX-HPL pathway proteins was confirmed [32]. To investigate the upstream regulatory network of *GmHPL*, we employed the yeast one-hybrid technique to screen for *GmHPL* transcription factors. After screening and one-to-one verification, two genes showed positive clones, *Glyma.05G063500* and *Glyma.13G286700*, named *GmERF36* and *GmILR3*. The dual-luciferase assay verified the results of the yeast one-hybrid experiment and showed that *GmERF36* and *GmILR3* suppressed the protein level of GmHPL. According to the SMART database, the GmERF36 protein contains an AP2/ERF domain, which has been shown in various proteins to be necessary and sufficient to bind to the GCC-box [33] and is possibly involved in the plant–pathogen interaction pathway. The Arabidopsis ortholog of *GmERF36*, *ERF102* (ERF5), a member of AP2/ERF subgroup IXb, is involved in plant pathogen resistance [34,35]. It has been reported that ERF102 (ERF5) and ERF103 (ERF6) are closely related, so the double mutant *erf102/erf103* was generated and infected with *V. longisporum*, which displayed significantly higher susceptibility compared with the wild type [36]. The AP2 domains of GmERF36, ATERF5, and ATERF6 show strong similarity (Figure S4). The GmILR3 protein contains a HLH domain, and the Arabidopsis ortholog of *GmILR3*, *ILR3* (*bHLH105*) is involved in iron homeostasis and plays an essential role in plant growth and abiotic and biotic stresses [37,38,39]. In Arabidopsis, ILR3 is induced in response to a wounding pathogen and the mutant *ilr3-2* showed significantly lower infection levels under Fe deficiency [38]. The HLH domains of GmILR3 and ATbHLH105 exhibit strong similarity (Figure S5). Later, Xing et al. revealed that a bacterial effector protein, AvrRps4, alleviates the degradation of bHLH115 and ILR3 to help pathogen colonization by iron acquisition [39]. Moreover, ILR3 confers resistance to chilling or high-light stress in plants by regulating iron homeostasis [37,40]. The present study indicated that GmHPL modulated the soybean defense response through the transcription factors GmERF36 and GmILR3.

It has been reported that *HPL* genes are induced by various stimuli, such as wounding, exogenous JA or MeJA, insects, and pathogens [23,41,42,43]. Different *HPLs* exhibit distinct expression trends in response to pathogens. After 4 h of *Botrytis cinerea* treatment, the expression level of the cucumber *HPL* gene reached its maximum [44]. After tobacco mosaic virus (TMV) infection in tobacco leaves, five *NtHPL* genes were upregulated, one *NtHPL* gene was downregulated, and three genes showed no significant differences in expression levels [45]. It was speculated that these *NtHPL* genes in tobacco may have different functions. Our qRT-PCR results showed that the expression pattern of *GmHPL* in soybean differed from those in cucumber and tobacco in response to pathogen stress.

After *C*. *truncatum* treatment, *GmHPL* and *GmERF36* initially displayed inverse expression patterns within 24 h: *GmERF36* was upregulated while *GmHPL* was downregulated. Subsequently, both genes were upregulated simultaneously, peaking at 48 h before decreasing. This result indicates that, during the initial 72 h post-fungal infection, *GmERF36* accumulation was initiated within the first 24 h, binding to the *GmHPL* promoter and inhibiting its transcription and translation. Afterwards, *GmERF36* continued to accumulate but its suppression of was relieved *GmHPL*, leading to increased *GmHPL* expression and the initiation of its function until the 72 h time point. However, *GmILR3* exhibited an expression pattern identical to that of *GmHPL*, decreasing initially and then increasing, which contradicts the LUC assay results and suggests that *GmILR3* was not involved in regulating *GmHPL* expression under pathogen treatment. GmILR3 may function in other stress responses or participate in processes such as post-transcriptional modification or complex formation with other proteins. Our findings show that the response of GmHPL to pathogen stress is a complex process. It is not only regulated by GmERF36 but also involves other transcription factors, and the underlying regulatory mechanism is dynamic over time. Further work (e.g., promoter mutagenesis to map binding sites or ChIP-qPCR validation) will be performed to reveal the mechanism.

## 4. Materials and Methods

### 4.1. Plant Materials and Growth Conditions

The soybean cultivar Williams82 (W82) was obtained from the National Center for Soybean Improvement at Nanjing Agricultural University. Soybean seeds were sown in pots and cultivated in a greenhouse (temperature at 25 °C/23 °C with a 16 h/8 h day/night photoperiod, 70% relative humidity) until the flowering and maturity stage. DNA was extracted from the W82 leaves.

### 4.2. Yeast One-Hybrid Analysis

The promoter sequence of *GmHPL* was obtained from the soybean genome data for *Glycine max Wm82.a4.v1* on the Phytozome website (http://phytozome-next.jgi.doe.gov/ accessed on 11 April 2024). Using DNA as the template, restriction enzyme sites KpnI and XhoI were added to both ends. After PCR amplification, the sequence was ligated into the pAbAi vector and transformed into *Escherichia coli*. The bacteria were spread on a plate and single clones were selected from the plate. After sequencing to confirm the correct sequence, the bait expression vector pAbAi-GmHPL-pro was constructed.

To perform the self-activation assay, the recombination vectors were transformed into yeast competent cells and plated on SD/-Ura agar plates containing different concentrations of Aureobasidin A (AbA, 100, 200, 300, 500, 700, and 900 ng/mL). Yeast one-hybrid screening was conducted using non-self-activating promoter sequences with a pre-established soybean whole-tissue yeast cDNA library. After preliminary and secondary screening, the one-to-one verification of positive clones was performed. The full-length CDSs of positive genes were cloned and inserted into the pGADT7 vector via the EcoRI restriction site to construct recombinant plasmids. Subsequently, these plasmids were co-transformed with pAbAi-GmHPL-pro into yeast competent cells for yeast one-hybrid pairwise validation.

### 4.3. Dual-Luciferase Reporter Assay

According to the yeast one-hybrid results, *Glyma.05G063500* and *Glyma.13G286700* interacted with the *GmHPL* promoter. Therefore, the full-length CDSs of *Glyma.05G063500* and *Glyma.13G286700* were cloned and inserted into the XhoI-digested pFGC5941 vector via homologous recombination to serve as effectors. The region 2759 bp upstream of the *GmHPL* CDS was cloned and inserted into the BamHI-digested pCM1300-Dual-LUC vector via homologous recombination to serve as the reporter.

Tobacco plants were cultivated in an artificial climate chamber under controlled conditions: temperature maintained at 25 °C/23 °C with a photoperiod of 16 h light/8 h darkness and 70% relative humidity. When the tobacco plants had developed 5–6 leaves, the Agrobacterium suspension stored at −80 °C was mixed with YEP liquid medium containing 50 µg/mL kanamycin and 25 µg/mL rifampicin at a ratio of 1:1000 (v:v). The mixture was incubated overnight at 28 °C with shaking at 200 rpm until the OD600 reached 1.0–1.2. After centrifugation at 5000 rpm for 5 min, the supernatant was discarded, and an appropriate amount of resuspension buffer was added (80 µL of 20 mg/mL AS, 400 µL of 1 M MgCl_2_, 400 µL of 1 M MES, adjusted to a final volume of 40 mL with ddH_2_O).

The resuspended pFGC5941-Glyma.05G063500 or pFGC5941-Glyma.13G286700, pCM1300-GmHPL-pro-LUC, and p19 were mixed at a volume ratio of 1:1:1, while the control consisted of pCM1300-GmHPL-pro-LUC and p19 at a volume ratio of 1:1. The final OD600 was adjusted to approximately 0.3, and the mixture was left in the dark for 3–4 h. The bacterial suspension was then infiltrated into the fully expanded leaves of 4-week-old tobacco plants using a needleless syringe via the pressure infiltration method on the back.

After infiltration, the tobacco plants were first incubated in the dark for 24 h, followed by controlled conditions as mentioned above. Before fluorescence observation, D-luciferin potassium salt (Cat#4090, Yeasen Biotechnology Co., Ltd., Shanghai, China) was injected into the infiltrated leaf areas. After incubation in the dark for 5 min, the leaves were removed and placed upside down in a plant live imaging system (Berthold LB985) to check fluorescence. Simultaneously, the luminescence values of LUC and REN were measured following the protocol of the Dual-Luciferase Reporter Assay Kit (11402ES60, Yeasen Biotechnology Co., Ltd., Shanghai, China). Each experimental group included 12 biological replicates, with each sample containing 1 cm diameter leaves.

### 4.4. Treatment with C. truncatum

The preserved *C*. *truncatum* was taken out from the −80 °C freezer and inoculated onto potato dextrose agar (PDA) plate medium. After culturing at 25 °C for 5 days, several mycelial plugs (5 mm in diameter) were obtained using a punch and inoculated into the potato dextrose broth (PDB) plants. When soybeans had developed 5–6 trifoliate leaves, the mycelia of *C*. *truncatum* were cultured in a shaking incubator at 25 °C and 120 rpm for 4–5 days. The mycelia were filtered, homogenized using a sterile blender, and diluted with sterile water to a concentration of 2 g/L. The suspension was evenly sprayed onto the surfaces of soybean leaves. Then, the treated plants were cultured at 25 °C, covered with plastic bags to keep the humidity above 70%.

### 4.5. Quantitative Real-Time PCR

After treatment with *C*. *truncatum,* soybean leaves were collected at 0, 12, 24, 48, and 72 h post-inoculation, with three biological replicates per time point. Each replicate consisted of leaves collected from three individual plants. Total RNA was extracted from soybean leaves using the DP419 kit (Tiangen, Beijing), following the instructions. Then, agarose gel electrophoresis was conducted to check the RNA quality. NanoDrop was used to measure the RNA concentration and A260/A280 (1.8–2.1) and A260/A230 (>2.0). Equal amounts of qualified RNA were used for reverse transcription. cDNA synthesis was performed using the ToloScript RT EasyMix for qPCR (#222107, Tolobio, Shanghai, China). For qRT-PCR, the 2×Q3 SYBR qPCR Master Mix (Universal) (#22204, Tolobio, Shanghai, China) was employed, with *GmActin* serving as the internal reference. The annealing temperature was set at 60 °C. Each sample was analyzed with three technical replicates, and relative expression levels were calculated using the 2^−ΔΔCt^ method. Statistical analysis was performed using Student’s *t*-test.

## 5. Conclusions

In the present study, by analyzing the upstream promoter region of the *GmHPL* gene, *cis*-elements such as MYB motifs, G-box motifs, ERE motifs, and W-box motifs were predicted, which were related to the stress response and helped to identify transcription factors. Using the *GmHPL* promoter as the bait, two transcription factors of *GmHPL* were identified, GmERF36 and GmILR3, through yeast one-hybrid library screening. A dual-luciferase assay verified the results of the yeast one-hybrid experiment and showed that GmERF36 and GmILR3 can inhibit the protein levels of GmHPL. Our qRT-PCR results showed that *GmERF36* accumulation was initiated within the first 24 h after *C. truncatum* treatment, binding to the *GmHPL* promoter and inhibiting its transcription and translation. Afterwards, *GmERF36* continued to accumulate but its suppression on *GmHPL* was relieved, leading to an increase in *GmHPL* expression until the end of the 72 h period. Our findings show that the response of *GmHPL* to pathogen stresses is a complex process. It is not only regulated by GmERF36 but also involves other transcription factors, and the underlying regulatory mechanism is dynamic over time.

## Figures and Tables

**Table 1:** Interacting proteins of *GmHPL* promoter in the yeast one-hybrid screening library.

Clone ID	Gene ID	Functional Annotation
1	Glyma.05G049900	AP2 domain
2	Glyma.08G297200	Whirly transcription factor
3	Glyma.10G186800	AP2 domain
4, 25, 32	Glyma.17G124100	AP2 domain
5	Glyma.13G286700	Helix–loop–helix DNA-binding domain
6, 14, 18	Glyma.05G063500	AP2 domain
7	Glyma.09G034500	Transcription factor bHLH148
8	Glyma.17G035200	Response to hydrogen peroxide, response to oxidative stress, response to salt stress
9	Glyma.07G048200	B3 DNA-binding domain
10	Glyma.14G063400	Myb-like DNA-binding domain
11	Glyma.19G061600	Myb-like DNA-binding domain
12	Glyma.10G146300	bHLH149-like
13	Glyma.19G178500	Histone-like transcription factor (CBF/NF-Y) and archaeal histone
15	Glyma.13G290100	Myb-like DNA-binding domain
16, 17	Glyma.06G266800	Helix–loop–helix DNA-binding domain
19	Glyma.09G131400	Myb-like DNA-binding domain
20	Glyma.14G020100	AP2 domain
21	Glyma.08G142400	WRKY DNA-binding domain
22	Glyma.04G008900	GATA zinc finger
23	Glyma.03G127800	VQ motif
24	Glyma.02G016100	AP2 domain
26	Glyma.14G086500	Myb-like DNA-binding domain
27	Glyma.18G181300	Myb-like DNA-binding domain
28, 56	Glyma.02G012700	bZIP transcription factor
29	Glyma.09G017400	MYB-CC type transfactor, LHEQLE motif
30	Glyma.05G148800	K-box region
31	Glyma.12G039900	NAC domain
33	Glyma.15G077100	AP2 domain
34	Glyma.04G177300	Myb-like DNA-binding domain
35	Glyma.14G139700	bZIP transcription factor
36	Glyma.11G142900	Myb-like DNA-binding domain
37	Glyma.17G075200	Helix–loop–helix DNA-binding domain
38, 62	Glyma.17G237900	Myb-like DNA-binding domain
39	Glyma.08G215300	bHLH25-like
40	Glyma.12G226600	AP2 domain
41	Glyma.02G161100	bZIP transcription factor
42	Glyma.08G325900	GRAS domain family
43	Glyma.04G145000	Histone-like transcription factor (CBF/NF-Y) and archaeal histone
44	Glyma.08G274200	Helix–loop–helix DNA-binding domain
45	Glyma.07G057400	WRKY DNA-binding domain
46	Glyma.13G189400	Histone-like transcription factor (CBF/NF-Y) and archaeal histone
47, 52	Glyma.15G003300	WRKY DNA-binding domain
48	Glyma.12G136300	Helix–loop–helix DNA-binding domain
49	Glyma.03G252100	Whirly transcription factor
50	Glyma.03G240000	Helix–loop–helix DNA-binding domain
51	Glyma.16G023000	Myb-like DNA-binding domain
53	Glyma.18G105800	SRF-type transcription factor (DNA-binding and dimerization domain)
54	Glyma.10G013300	bZIP transcription factor
55	Glyma.04G103900	AP2 domain
57	Glyma.17G145300	AP2 domain
58	Glyma.13G236500	AP2 domain
59	Glyma.20G175000	Transcription factor UPBEAT1
60	Glyma.15G261300	Histone-like transcription factor (CBF/NF-Y) and archaeal histone
61	Glyma.18G159900	AP2 domain
63	Glyma.14G185800	WRKY DNA-binding domain
64	Glyma.03G135800	HSF-type DNA-binding domain

## Data Availability

The original contributions presented in this study are included in the article/Supplementary Materials. Further inquiries can be directed to the corresponding author.

## Supplementary Materials

The following supporting information can be downloaded at https://www.mdpi.com/article/10.3390/plants15010054/s1. Figure S1: Self-activation test of the *GmHPL* gene promoter in the yeast one-hybrid assay. Figure S2: The results of the yeast one-hybrid library screening. Figure S3: The LUC and REN activity of GmHPL-pro-LUC (GmHPL-pro), 35S-GmERF36 and GmHPL-pro-LUC (GmERF36 + GmHPL-pro), and 35S-GmILR3 and GmHPL-pro-LUC (GmILR3 + GmHPL-pro). Each experimental group included 12 biological replicates and three technical replicates, with each sample containing 1 cm diameter leaves. ** indicates significant differences at *p* < 0.01. Figure S4: Amino acid sequence alignment of GmERF36 protein in soybean and its ortholog genes in Arabidopsis. The red underlines indicate the AP2 domains. Figure S5: Amino acid sequence alignment of GmILR3 protein in soybean and its ortholog genes in Arabidopsis. The red underlines indicate the HLH domains.
