# Supplementary material for: Identification of Transcription Factors of GmHPL Involved in Modulating Pathogen Stresses in Soybean
Source: Plants (Basel). 2025 Dec 24;15(1):54. doi: 10.3390/plants15010054 (PMC12787735; doi:10.3390/plants15010054)
Supplement: Supplementary file 1 [file plants-15-00054-s001.zip › plants-4028947-supplementary.pdf]

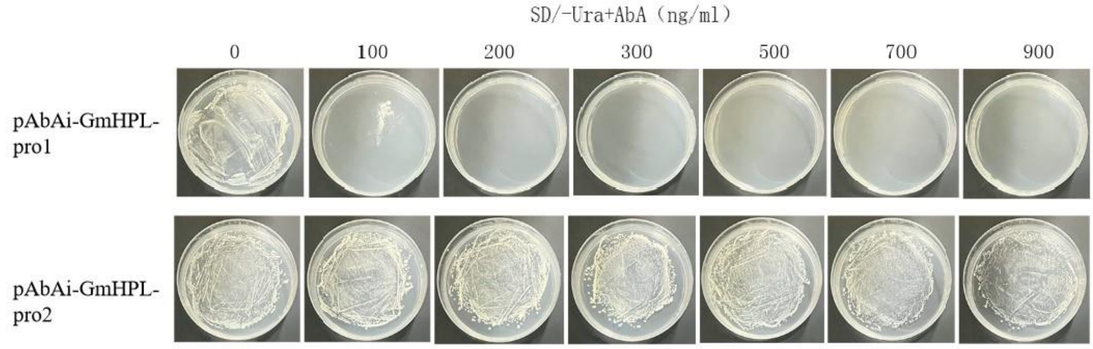

Figure S1. Self-activation test of the *GmHPL* gene promoter in the yeast one-hybrid assay. GmHPL-pro1 refers to the region from -1 to -1032 bp upstream of the *GmHPL* gene, and GmHPL-pro2 refers to the region from -1524 to -2759 bp upstream of the *GmHPL* gene.

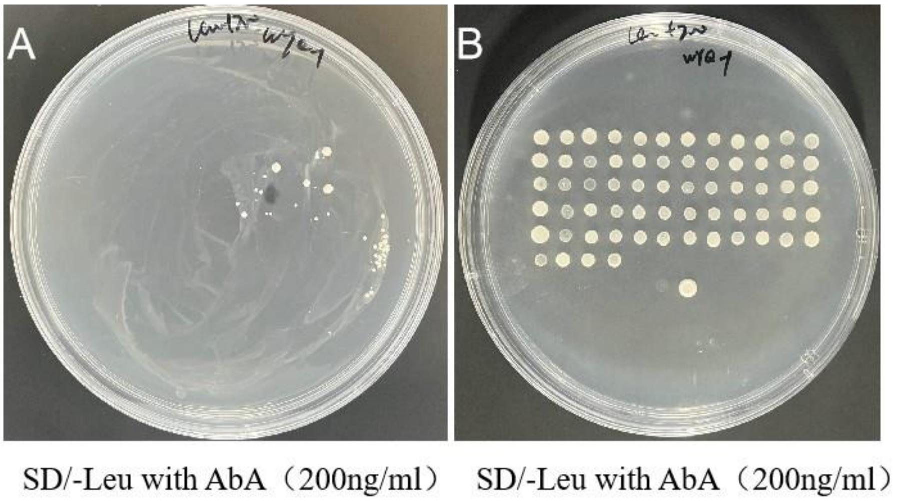

Figure S2. The results of the yeast one-hybrid library screening. (A) Partial results of the primary screening. (B) The results of the secondary screening.

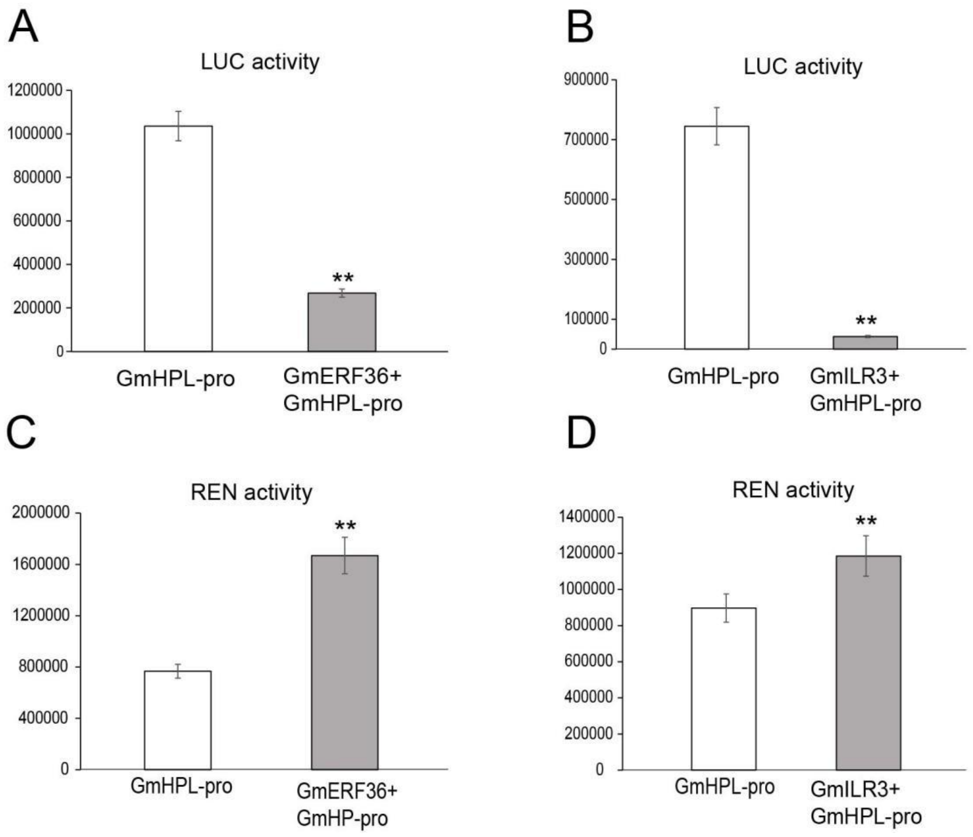

Figure S3. The LUC and REN activity of GmHPL-pro-LUC (GmHPL-pro), 35S-GmERF36 and GmHPL-pro-LUC (GmERF36 + GmHPL-pro) and 35S-GmILR3 and GmHPL-pro-LUC (GmILR3 + GmHPL-pro). Each experimental group includes 12 biological replicates and three technical replicates, with each sample containing 1 cm in diameter leaves.** indicates significant differences at $p < 0.01$.

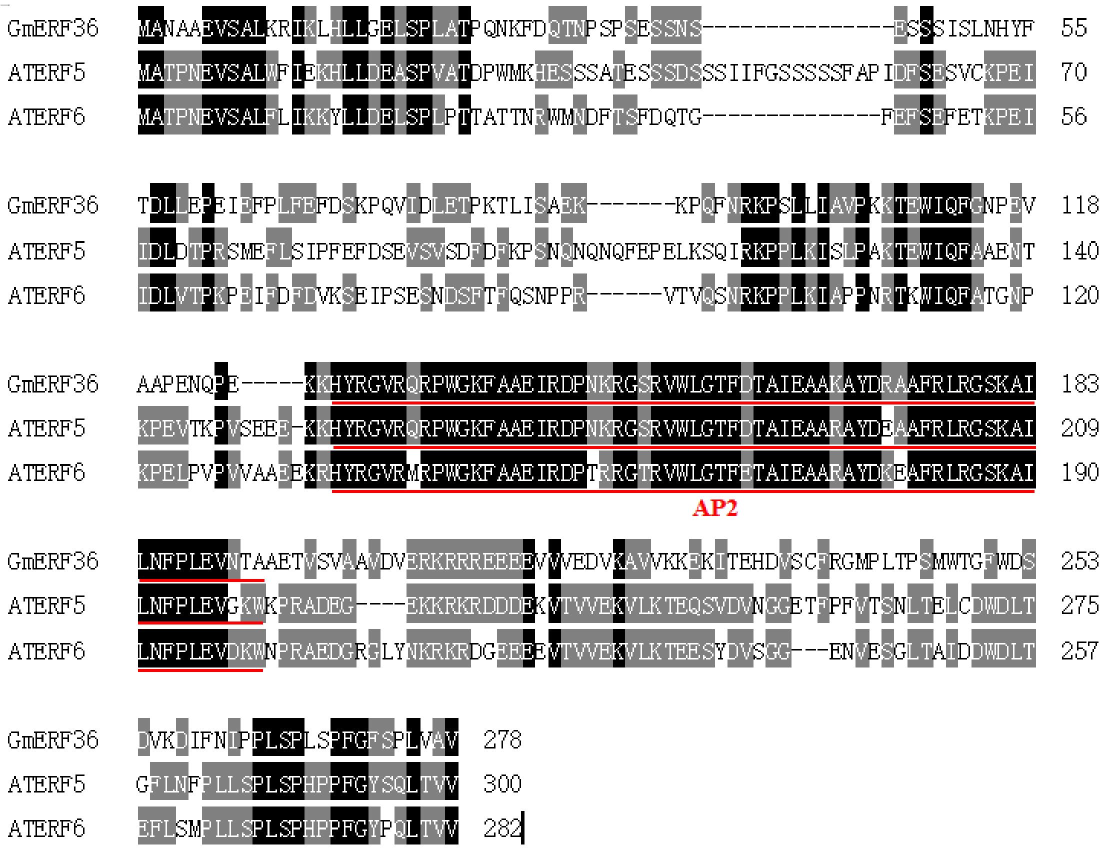

Figure S4. Amino acid sequence alignment of GmERF36 protein in soybean and it's orthologs genes in Arabidopsis. The red underlines represent the AP2 domains.

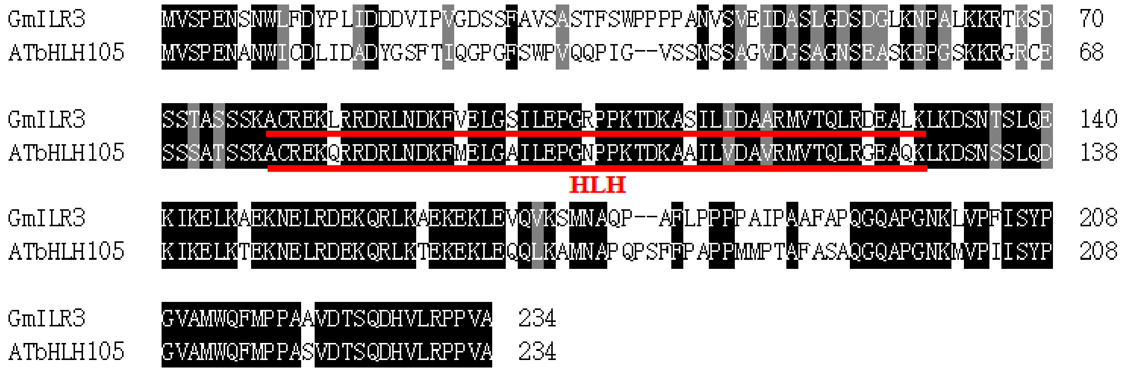

Figure S5. Amino acid sequence alignment of GmILR3 protein in soybean and it's orthologs gene in Arabidopsis. The red underlines represent the HLH domains.
